# Using Effective Subnetworks to Predict Selected Properties of Gene Networks

**DOI:** 10.1371/journal.pone.0013080

**Published:** 2010-10-08

**Authors:** Gemunu H. Gunaratne, Preethi H. Gunaratne, Lars Seemann, Andrei Török

**Affiliations:** 1 Department of Physics, University of Houston, Houston, Texas, United States of America; 2 Department of Biology and Biochemistry, University of Houston, Houston, Texas, United States of America; 3 Human Genome Sequencing Center and Department of Pathology, Baylor College of Medicine, Houston, Texas, United States of America; 4 Department of Mathematics, University of Houston, Houston, Texas, United States of America; Centre for Genomic Regulation (CRG), Universitat Pompeu Fabra, Spain

## Abstract

**Background:**

Difficulties associated with implementing gene therapy are caused by the complexity of the underlying regulatory networks. The forms of interactions between the hundreds of genes, proteins, and metabolites in these networks are not known very accurately. An alternative approach is to limit consideration to genes on the network. Steady state measurements of these *influence networks* can be obtained from DNA microarray experiments. However, since they contain a large number of nodes, the computation of influence networks requires a prohibitively large set of microarray experiments. Furthermore, error estimates of the network make verifiable predictions impossible.

**Methodology/Principal Findings:**

Here, we propose an alternative approach. Rather than attempting to derive an accurate model of the network, we ask what questions can be addressed using lower dimensional, highly simplified models. More importantly, is it possible to use such robust features in applications? We first identify a small group of genes that can be used to affect changes in other nodes of the network. The reduced effective empirical subnetwork (EES) can be computed using steady state measurements on a small number of genetically perturbed systems. We show that the EES can be used to make predictions on expression profiles of other mutants, and to compute how to implement pre-specified changes in the steady state of the underlying biological process. These assertions are verified in a synthetic influence network. We also use previously published experimental data to compute the EES associated with an oxygen deprivation network of *E.coli*, and use it to predict gene expression levels on a double mutant. The predictions are significantly different from the experimental results for less than 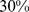 of genes.

**Conclusions/Significance:**

The constraints imposed by gene expression levels of mutants can be used to address a selected set of questions about a gene network.

## Introduction

Living systems are typically able to maintain their physiological state under environmental changes and isolated genetic mutations [1]. This robustness, referred to as homeostasis [2] or canalization [3], [4], is achieved through feedback within highly connected regulatory networks of genes, proteins and metabolites [5]–[10]. For example, an action that reduces the expression of one gene may cause coordinate changes in other nodes to leave the physiological state unaffected. If a genetic mutation blocks one pathway, other avenues on the associated network may take its place. Unfortunately, this systemic stability often makes it difficult to eliminate defects in a biological network, as evidenced by the surprising lack of efficacy of many drugs that were designed to act on single molecular targets [11], [12]. The coupling can also lead to side effects from medications. For example, anti-inflammatory COX-2 inhibitors (*e.g.*, Vioxx) cause adverse cardiovascular effects due to a concomitant imbalance of the lipids prostacyclin and thromboxane A2, which lie on the same network [13]. Clearly, the most effective and least detrimental changes in a biological process are implemented by altering the system in its entirety. This task requires predictive mathematical models which can be constructed from experimental data. In this paper, we propose an approach for such a construction.

There are hundreds of genes, proteins, and other molecular participants associated with most biological processes. Gene regulatory networks model all interactions between these nodes. However, the forms of these dependencies, as well as kinetic parameters such as reaction rates and diffusion constants are, at best, only known approximately [14]. It is unlikely that gene regulatory networks which are sufficiently accurate to make quantitative predictions on the underlying biological processes will be available in the near future [15], [16].

Many approaches to reduce the complexity of regulatory networks have been proposed [5]. Small modules or network motifs [17], [18] associated with specific tasks have been identified. Boolean variables [19] can reduce the complexity, although the coarse-graining will limit predictability to qualitative characteristics such as bifurcations. In *gene influence networks* [14], [20], a gene, its transcript, and protein are represented by a single node, which is quantified by the expression level of the mRNA. Regulatory interactions between nodes of an influence network include actions mediated by other components in the network.

Consider an influence network containing 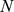 genes 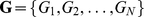; denote the expression level of the 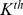 gene by 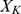 and the state of the network by 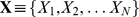. The influence network can be modeled by a set of ordinary differential equations 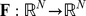


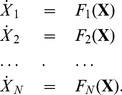
(1)Steady states of influence networks can be obtained from DNA microarray experiments [5]. However, most influence networks contain hundreds of genes; thus, even if 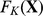 are assumed to have a simple (*e.g.*, linear) form [21], a prohibitively large number of microarray experiments will need to be conducted in order to compute 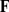. Moreover, gene expression levels in microarray experiments have large (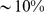) error bars; when 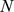 is large, the inversions needed to compute 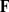 will exacerbate the uncertainty to a level which will make predictions difficult. One possibility is to only extract partial information on these networks through inference algorithms such as Network Identification by Multiple Regression [14], and Mode-of-action by Network Identification [22].

We propose an alternative approach. Rather than attempting to construct an accurate model of a gene network, we ask what questions on the network can be addressed (perhaps approximately) using low-dimensional and highly simplified effective models constructed from empirical data. What data would be needed for the construction? Will issues addressed through the approach be useful in applications?

We first note that genes in an influence network can be partitioned into strongly coupled subgroups or clusters. This partition can be made either using co-expression under genetic perturbations [23]–[25], or through the use of the Gene Ontology (GO) database (http://geneontology.org). Our main assumption is that the behavior of all nodes within a cluster can be controlled by imposing suitable changes in a small, specially chosen, subset of its members. The set could include genes that translate to transcription factors, and would hence influence many other genes [26], [27]. It may also include microRNAs within the cluster, each of which affect many genes through post-transcriptional regulation [28], [29], even though their fold induction on each gene is small.

Suppose we have partitioned the 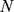 genes of the influence network into clusters, and identified a small number of genes/microRNAs from each cluster that can be used to control the expression levels of the remaining genes. Denote the set of these nodes by 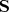. The number 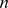 of nodes in 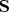 is much smaller than 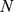. We will represent their expression levels by 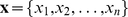, and re-index the variables so that the remaining expression levels are 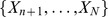. With the new ordering, we write the state of the network as 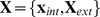 where we will refer to 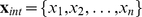 and 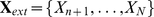, as “internal” and “external” variables respectively.

**Figure 1:**
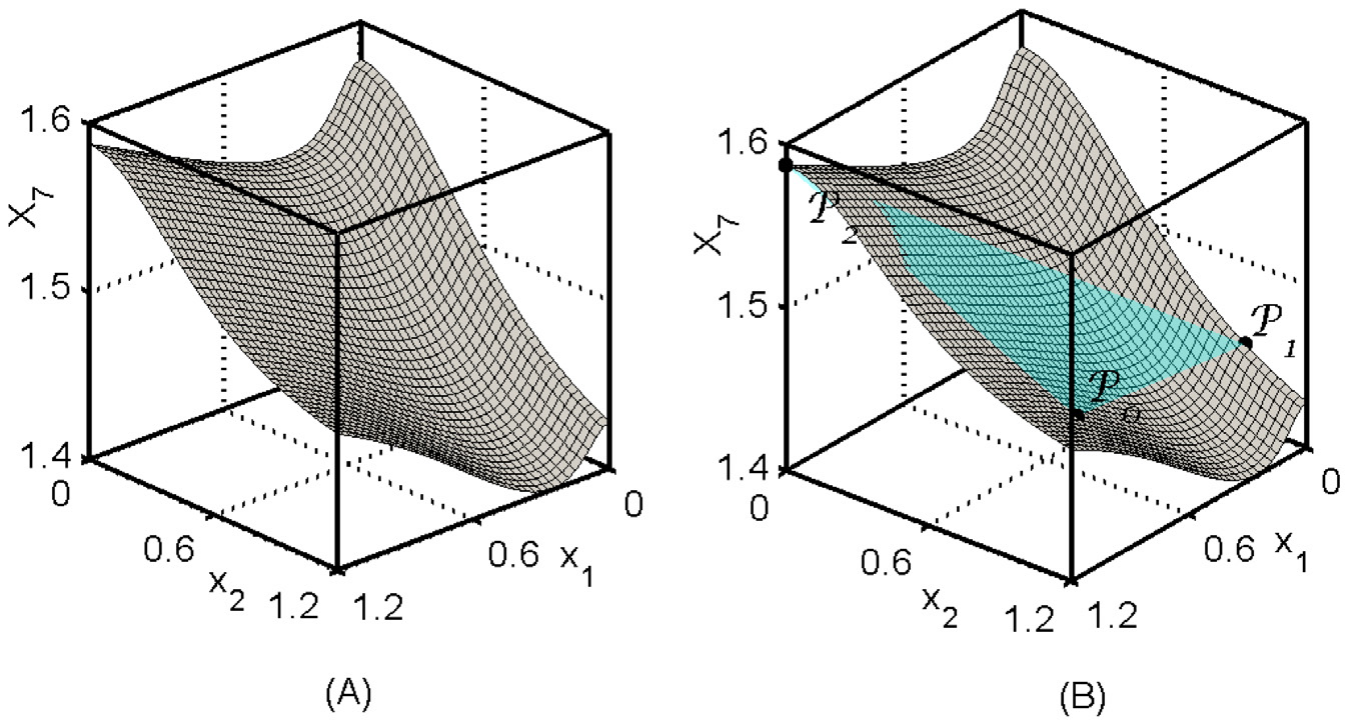
Example of an 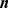-dimensional solution surface 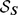 of (1). The example is chosen from the synthetic network introduced in the Results Section. (a) The surface shown represents the expression levels of the external variable 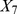 as the internal variables 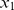 and 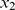 are modified. (b) The point 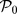 representing expression levels of the wildtype and points 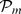, 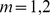 representing expression levels of single knockout mutants 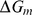 lie on this surface. The EES is defined so that its solutions lie on the unique 2-dimensional plane (blue) 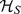 passing through 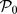, and 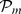, 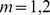. As can be seen, due to restrictions imposed on the EES, the surfaces 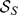 and 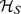 are close.

In this paper, we limit consideration to networks with steady state solutions. When external perturbations are made on genes within 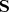, expression levels of the remaining genes at equilibrium are determined by Eqn. (1). These steady states lie on an 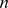-dimensional surface in 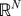, which we denote by 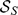. Figure 1(a) shows a schematic (2-dimensional) solution surface for the synthetic network introduced in the Results Section.

We make the following observations on solutions of the system 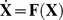. First, we assume that the unconstrained system has a unique stable steady state which we denote by 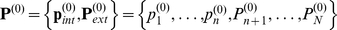. It satisfies the 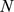 equations 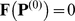. The point 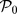 representing it lies on 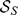. Next, consider the single knockout mutant 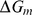 (assumed to be viable) obtained by knocking out the 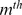 gene. Since 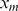 is set externally, the 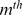 equation of (1) is no longer valid. The solution for the expression levels is obtained by solving the remaining 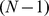 equations. We denote this equilibrium by 
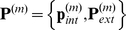
 with 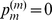, and represent it by 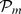. Since the equilibrium is associated with changes made within the set 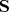, 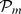 lies on 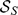. Consequently, 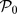 as well as 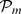 for 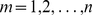 lie on 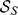, see Figure 1(b).

Our goal is to construct a system, referred to as the “effective empirical subnetwork” (EES), that can be computed using the gene expression levels of mutants discussed above, and whose equilibria are close to 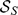. Observe that 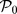 and the 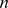 points 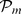 define a unique 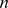-dimensional plane in 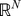, which we denote by 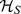. Figure 1(b) shows the surface for the example above. The EES describes the set 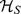 as parametrized by the internal variables. Since both 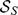 and 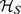 contain the points 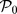, and 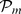, 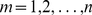), we expect them to be close in the region of interest.

Observe that the 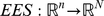 is a linear function determined by 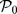, and 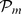, (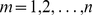), but is otherwise independent of 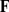. In particular, each 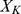 is a linear function of 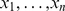. Since 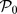 lies on 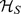


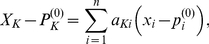
(2)for each 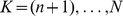. The coefficients 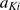 can be evaluated by noting that 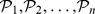 lie on 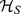. There is one additional complication, that we illustrate using the following example. Suppose we consider a mutant where only 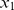 is externally set. The remaining expression levels of the steady state of this mutant are solved using the last 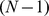 components of Eqn. (1). In particular, the expression levels 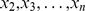 of the internal variables in this mutant depend on 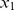. In general, the internal variables themselves depend on the gene expression levels whose values are externally imposed. Thus, we expect there to be relationships between the internal variables as well. As we show in the Methods Section, these dependencies can be assumed to take the form
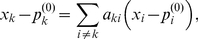
(3)for 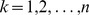.

**Figure 2:**
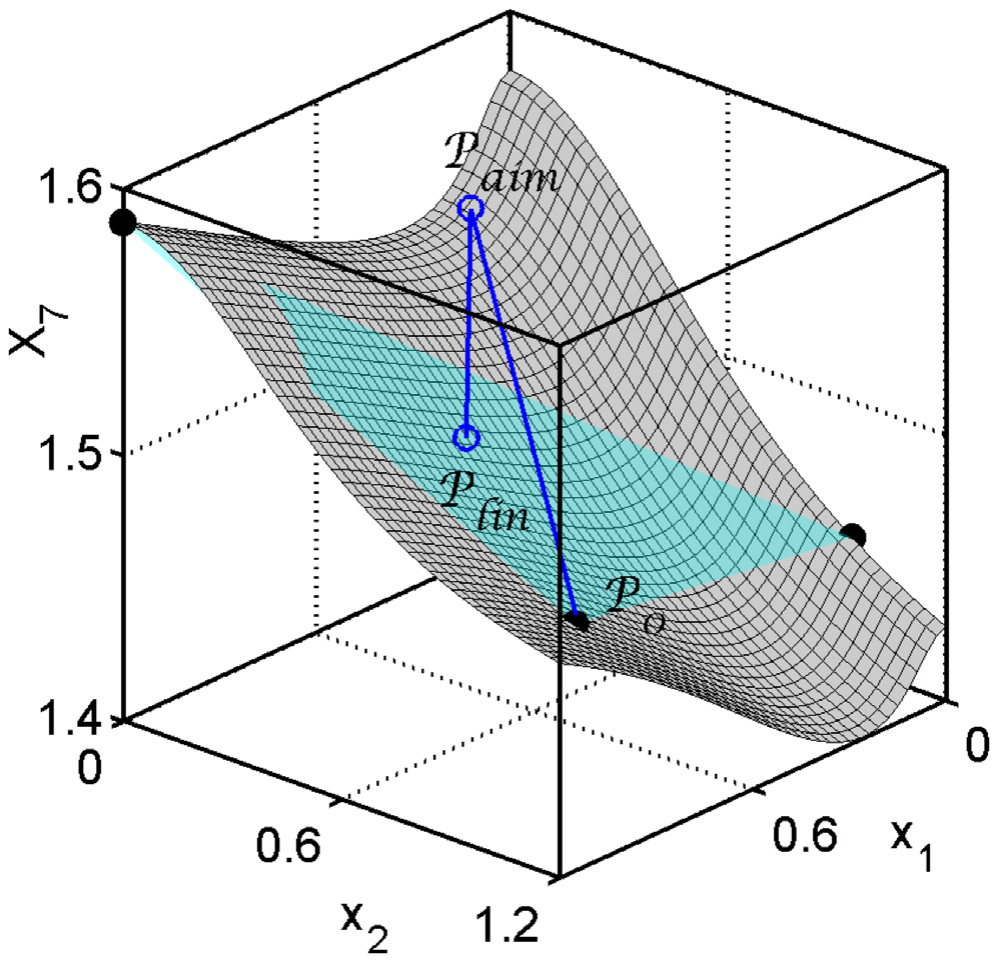
Moving the equilibrium from 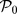 to 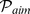 by implementing changes within 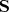. In general this is not possible because interactions between nodes force the equilibrium to remain on 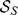. However, it is possible to compute 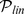, the point closest to 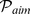 that can be reached by the EES. Due to the proximity of 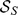 and 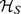, the point 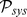 obtained by projecting 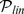 to 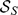 is close to 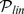. Thus, it is possible to pre-determine if the movement of the equilibrium forced by the changes made in 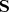 are acceptable.

We have thus implemented two significant simplifications. The original system 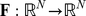 contained a large number (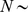
several hundred) of nonlinearly coupled variables. In contrast, the 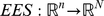 has a small number (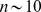) of internal variables, is linear, and can be constructed using the steady state solutions of the original system (wild-type) and 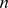 single knockout mutants. Clearly, the EES is not an accurate representation of the original network. The issue is whether there are questions about 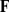 that can be addressed using the EES. As we show below, this is indeed the case due to geometrical constraints imposed on the solution surface. Specifically, the EES can be used to predict, approximately, the expression levels of all nodes in 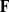, when external changes are made within 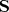; *e.g.*, double knockout mutants. The validity of the EES construction can be tested by comparing its predictions with microarray data from such mutants. More significantly, the EES can be used to compute how the equilibrium of the system can be moved from its initial state 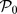 to a pre-specified set of expression levels defined by a point 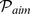, see Figure 2. Since 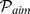 will, in general, not lie on the solution surface, it cannot be reached through changes within 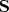. Instead, we can use the EES to compute 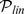, which is the closest point to 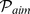 on the plane 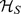, see Figure 2. Since the surfaces 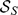 and 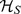 are close, changes imposed on the system by the external actions are expected to be close to those computed from the EES. Below, we verify this proximity in a synthetic influence network.

## Results

### A Synthetic Influence Network

In the model system, 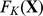 is a linear combination of sigmoidal Hill functions; specifically,
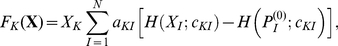
(4)where 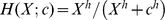 is the Hill function and the Hill index 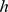 is chosen to be 2. The action of the 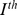 gene on the 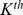 one is characterized by parameters 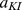 and 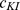, which are assumed to be independent of the state 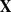 of the system. The action is activating if 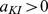 and inhibiting if 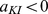. The system is constructed so that 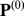 is a steady state of Eqns. (1). Numerically, we find that model systems defined by Eqns. (1) and (4), have at most one stable equilibrium. We suspect that this is due to restrictions imposed by the fact that the sign of each partial derivative 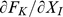 is independent of the state of the system.

In order to compute the solutions to the knockout mutant 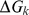, we set 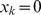, and solve the remaining equations of (1) as a nonlinear least squares problem. When the normalized residue fails to fall below 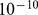, it is assumed that the corresponding solution does not exist.

**Figure 3:**
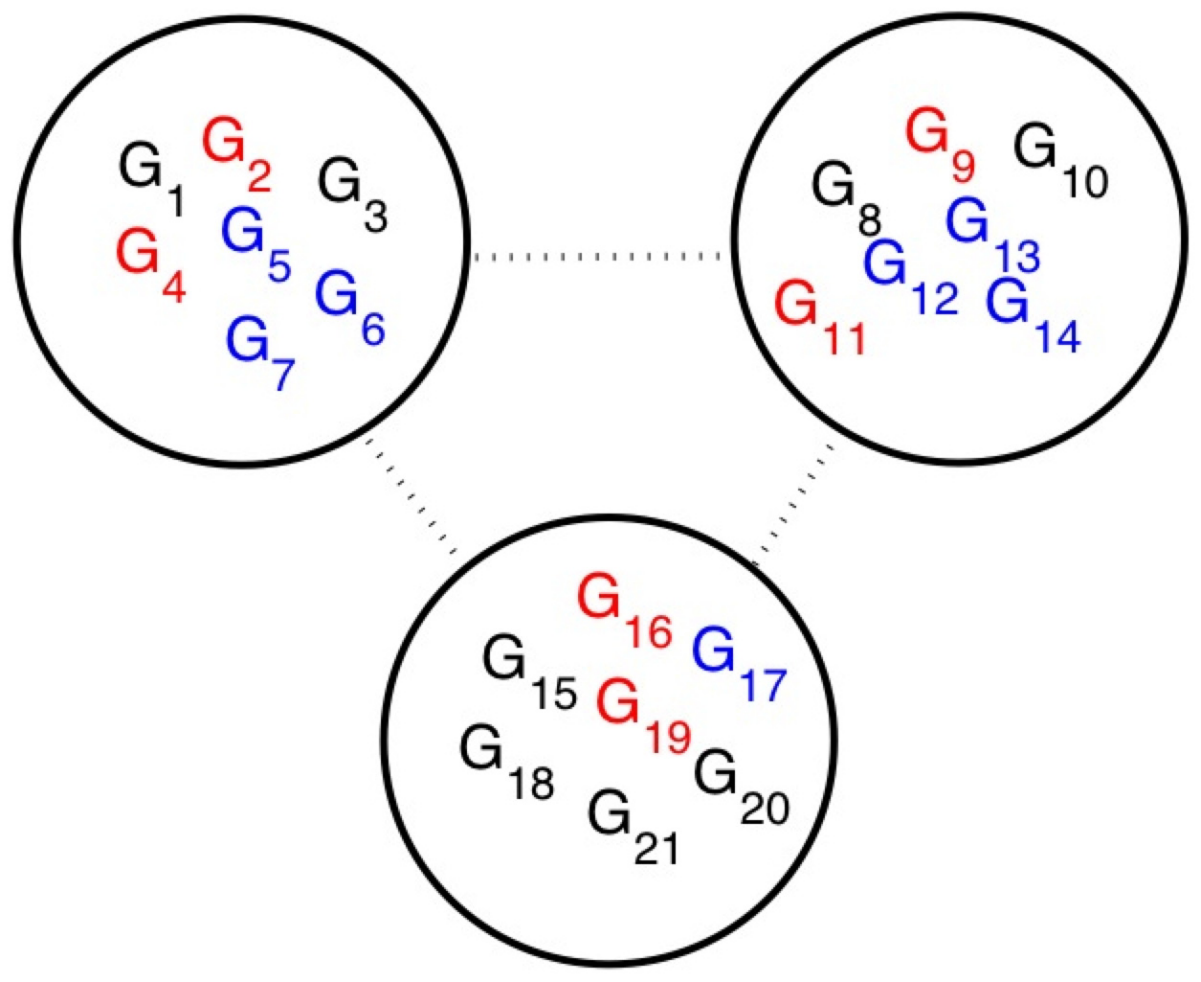
A schematic of the synthetic network. The 21 genes in the system consists of 3 groups, each with 7 genes. Genes within a cluster are coupled by interactions whose intensity is chosen randomly. Genes between clusters are weakly coupled. The “mutants” 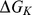 shown in black are not viable; i.e., the corresponding set of equations do not have a solution. Genes shown in red are used to construct the effective empirical subnetwork.

We report on a model system containing 21 nodes and shown schematically in Figure 3. We start with the three subnetworks, each of size 7. The vector 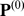 for each of these subgroups consists of random entries between 0.5 and 1.5, and the matrix 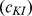 contains random values between 0 and 2. The entries of the Jacobian of the system given by Eqns. (1) and (4) at 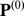 are 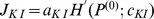; thus 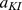 can be computed for a given set of 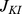
's. Since we require 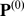 to be stable, we insist that all eigenvalues of the Jacobian be negative. This is guaranteed by starting with a diagonal matrix with negative entries and performing a (random) orthonormal transformation. Once three such subnetworks are computed, their nodes are coupled by sparse, weak interactions. Each node in a subnetwork is coupled to only one from each of the other subnetworks, and the mean coupling strength is chosen to be 0.1 of the average coupling within subgroups.

The EES is to be constructed using the expression levels of single knockout mutants. As illustrated in Figure 3, mutants 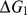, 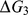, 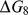, 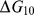, 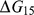, 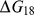, 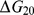, and 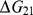 in our example are not viable; *i.e.*, when the corresponding 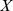 is set to zero, the system (1) does not have a solution. The subset on which to construct the EES can contain any of the other nodes. In the work reported here, 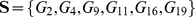 (genes marked in red in Figure 3). The variables 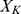, 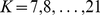 are re-indexed as described before. The 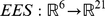 is computed using the expression levels of all 21 genes at 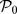, 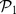, 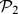, 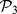, 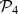, 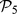 and 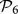.

In order to illustrate the proximity of 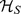 to 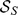, we use the following example, see Figure 1. Since we need to reduce the dimensionality for visualization, we fix the expression levels of (the re-indexed genes) 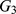, 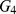, 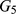, and 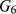 at their values at 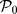; for our model, 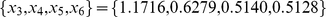. For each pair of values for 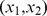, we solve the model system (1) for the remaining 15 expression levels. These solutions lie on 2-dimensional surface in 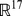. The gray surface of Figure 1 is 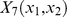. The 2-dimensional plane 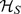 of the EES contains points 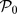, 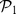, and 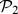.

Next, we compare expression levels of double knockout mutants predicted by the EES with the corresponding solutions of the model system (1). The 14 viable double knockout mutants of the system are 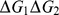, 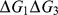, 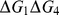, 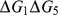, 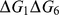, 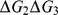, 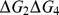, 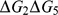, 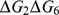, 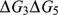, 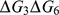, 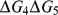, 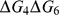, and 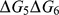. In each case, the expression levels of the 4 remaining nodes in 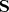, and the 15 nodes outside of 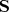 are compared. We differentiate between these two groups.

Results for the first group (genes in 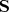) are as follows. Of the 56 comparisons, 46 EES predictions are within 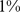 of the expression levels computed from (1), 3 others are between 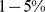, and 3 between 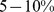. Results for the second group (genes outside of 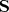) are as follows. Of the 210 expression levels to be compared, 170 EES predictions are within 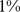 of the expression levels computed from (1), 30 more are between 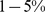, and 7 others are between 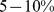.

We finally demonstrate how the equilibrium of the system can be moved (near) to a pre-specified set of expression levels. The original equilibrium of our example is 
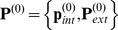
, with
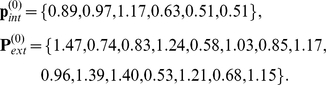
We want to find out how the expression levels of genes in 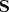 need to be changed so that the system moves to, or as close as possible to, a pre-specified set of expression levels for all genes. As an example, we attempt to change the equilibrium of the system to 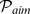 (see Figure 2) given by 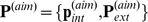, where
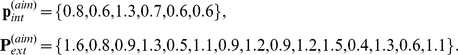
Since we have computed the EES, we can calculate the projection 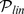 of 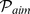 on 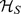. It is given by 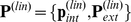, where
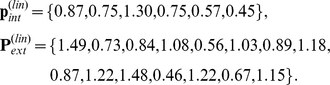
Finally, we use the model system Eqns. (1) and (4) to compute the external variables when internal variables are fixed at 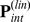. It is found to be 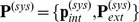, where 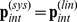, and
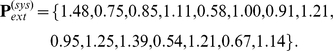
The Euclidean distances between the points are 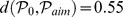, 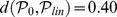, 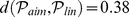, and 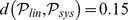. Thus, we attempted to move the equilibrium from 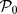 to a point 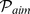 that was a distance 0.55 away, but were only able to move it on 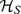 to a point 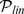, which is a distance 0.40 away from 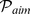. However, 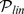 is only a distance 0.15 from the point 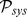, which is the solution of the original system when expression levels of the internal variables are set to 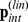. We have found that 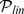 and 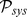 are close in studies of several model systems and for many points 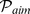. Thus, the EES can be used to pre-determine, approximately, the equilibrium of the original network when changes made within 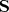.

### Transcriptional Regulatory Network in *E.coli*

The EES can be constructed using microarray data from the wildtype and single knockout mutants of genes in 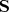. It can then be used to predict gene expression levels of other mutants. This observation is of interest due to the availability of previously published data on an oxygen deprivation network in *E.coli* [30], [31]. Ref. [32] reports gene expression levels in the wildtype and in single knockout mutants of key transcriptional regulators in the oxygen response, namely 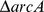, 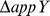, 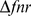, 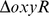, and 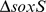, as well as in the double knockout mutant 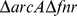, in aerobic and anaerobic glucose minimal medium conditions. Since the oxygen deprivation network is not fully active under aerobic conditions, we focus on the behavior of *E.coli* under anaerobic conditions.

It should be noted that gene expression levels in *E.coli* are unlikely to be in a steady state; rather, the expression levels reported in Ref. [32] are averages from a group of cells in various stages in the cell cycle. The analysis in this Section assumes that the computation of the EES and its predictions are valid for these averages. Preliminary results from our current work on systems exhibiting circadian rhythms validate this assumption.

We construct 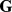 as follows. In the Gene Ontology classification assigned by Affymetrix, the five genes 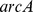, 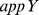, 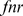, 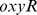, and 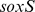 have a common term “GO:0006355, Regulation of transcription, DNA-dependent.” Moreover, this is the only common classification for the five genes. We choose 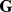 to be the set of all genes carrying this term. The full list of 299 genes is given in Supporting Information S1.

The data set GSE1121 of the GEO site (www.ncbi.nlm.nih.gov) [32] provides gene expression levels for four replicates of the wildtype and three each for the mutants. The replicates are used to estimate the mean and standard deviation for the expression levels of each gene in 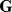, see Supporting Information S1. Since the EES is linear, we rescale the expression levels of each gene by its (mean) value in the wildtype. Table 1 gives these rescaled expression levels for the internal variables 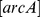, 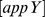, 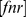, 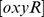, and 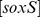 under anaerobic glucose minimal medium conditions.

**Table 1:** Normalized gene expression levels in the wildtype and mutants.

	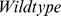	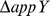	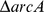	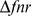	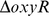	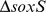	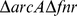
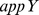	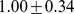	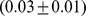	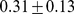	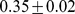	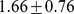	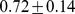	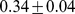
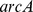	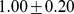	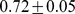	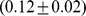	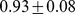	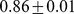	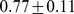	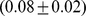
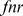	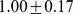	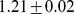	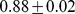	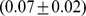	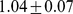	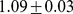	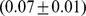
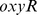	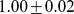	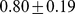	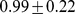	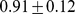	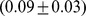	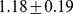	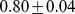
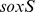	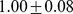	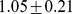	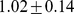	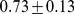	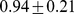	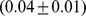	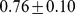

Rescaled expression levels of 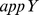, 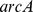, 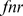, 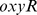, and 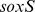 in the wildtype *E.coli*, single knockout mutants, and the double knockout mutant 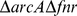 under anaerobic glucose minimal medium conditions. The data have been rescaled by the mean value of the expression levels in wildtype cells. The mean and standard errors are calculated from the replicates given in the data set GSE1121 of the GEO site www.ncbi.nlm.nih.gov. The values in parentheses are set to zero in computing the EES.

Note that error estimates for the expression levels of several genes is large. This is the reason that a reduced network is essential in order to make useful predictions. Second, as seen from Table 1, reported expression levels of the gene 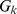 in the mutant 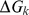 is non-zero. Presumably, what is measured are non-functional analogs of the corresponding genes. In calculating the EES, we set these expression levels (shown in parentheses in Table 1) to zero.

The component of the EES for the internal variables is
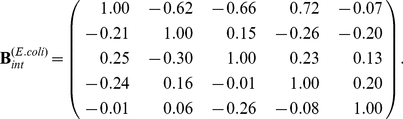
(5)The next step is to compute the EES predictions for 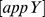, 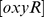, and 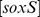 in the double knockout 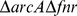. This is done using the matrix (5) and setting 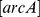 and 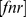 to zero. Expression levels of the remaining genes in 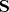, predicted using the EES, are 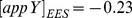, 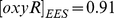, and 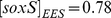. We need to determine, at a 5% level of confidence, if these predicted values are consistent with those from the replicates of the double mutant. The comparison is made using the 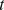-test (ttest in MATLAB, The Mathworks, Inc.), and it is found that the null hypothesis, that experimental data comes from a (normal) distribution with mean equal to the computed gene expression level, is rejected at the 5% level only for *appY*.

**Figure 4:**
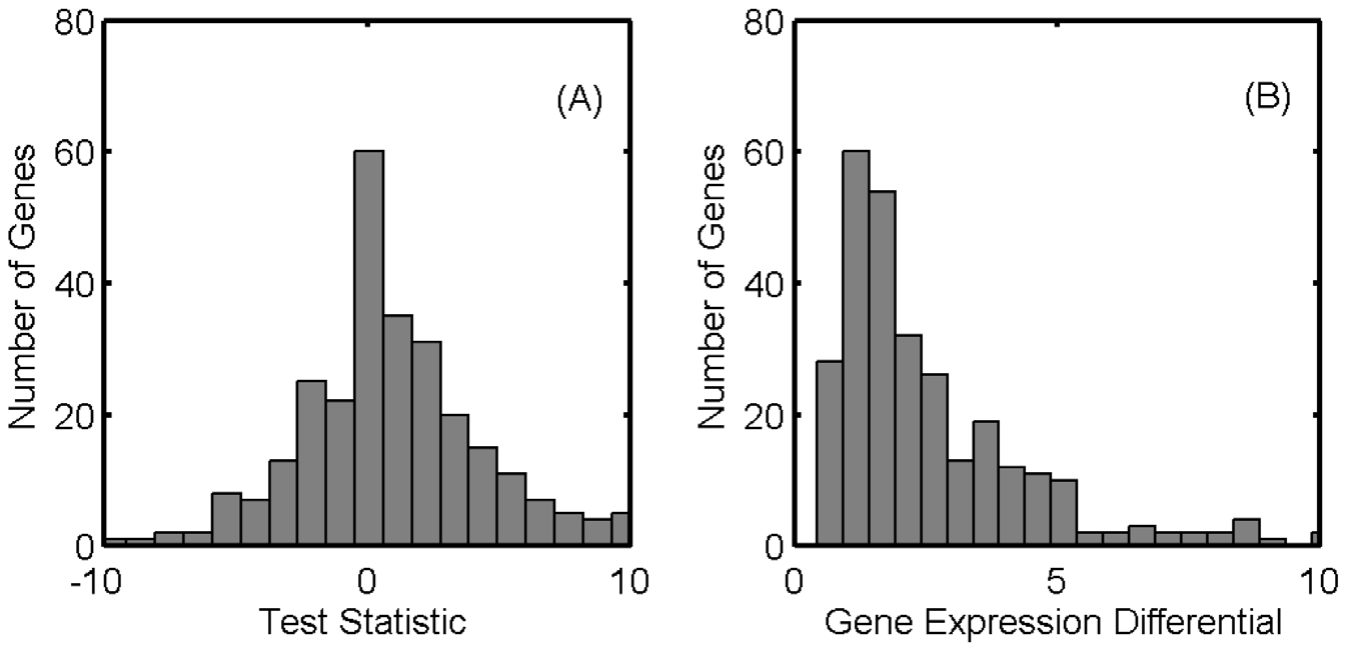
Comparison of EES predictions with experimental gene expression levels of 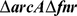. (a) The histogram of the test statistic of the Student's distribution (two degrees of freedom) for the 299 genes chosen for the study. (b) The good agreement in (a) is not due to lack of variation in the gene expression levels between the wildtype and mutants. The histogram shows the largest differential expression level of mutants, normalized by the standard deviation for the wildtype (computed from the four replicates given in the data set GSE1121 of the GEO database [32]).

Next, we implement the analysis for genes outside of 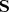. The null hypothesis cannot be rejected at the 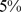 level for 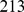 of the 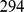 genes. The three experimental values of the expression level of each gene in the double knockout, the corresponding predictions of the EES, and the test statistic 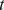 are given in Supporting Information S1. Since the Student's distribution associated with the comparison has two degrees of freedom, the null hypothesis is rejected when 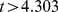. The histogram of the test statistic for the 299 genes is shown in Figure 4(a). In Supporting Information S1, we highlight the genes for which the null hypothesis is rejected. We emphasize that, unlike in many prior studies whose assertions are limited to whether genes in mutants are up/down regulated, our predictions are quantitative.

The proximity of the predicted and experimental values is not due to a lack of variability in the expression levels of genes in 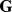. We verify this by computing the differential expression of genes in the mutants. Figure 4(b) shows the histogram of the largest deviations from the wildtype, normalized by the standard deviation (between replicates) in the wildtype. Expression levels of over half the genes deviate by more than 2 standard deviations.

## Discussion

An accurate model of the gene regulatory network associated with a hereditary disease can be used to compute the most effective and least detrimental treatment to prevent its onset. Unfortunately these networks contain hundreds of genes, proteins, and other molecules whose interactions are only partially known [5], [8]–[10]. It is unlikely that detailed models of such networks will be available in the near future. The question raised in the paper is whether information needed to move the steady state of a network can be deduced from an analysis of highly simplified, empirically determined models. The data used for analysis is obtained from microarray experiments.

Our approach is as follows. We first identify a (relatively) small set 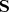 of 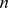 nodes (internal variables) in the influence network which can be used to affect the remaining genes. (Mathematically, for each external variable 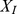, we require one or more of 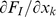, where 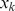 are the internal variables, to be non-vanishing.) Next, we limit consideration only to steady states of the network as internal variables are modified. Finally, this solution surface 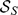 is approximated using the unique 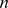-dimensional plane 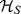 defined by the gene expression levels of the wildtype and the 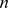 single knockout mutants in 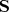; the model system whose solutions lie on the plane is the EES.

Some genes may be critical in the sense that the organism may not be viable when they are knocked out. There were similar nodes (shown in black in Figure 3) in our synthetic model. In our approach they cannot be used as internal nodes. However, if they need to be utilized, the EES can be computed using heterozygous mutants or those where the expression level is up/down regulated to a value other than zero through, for example, transfection [33], [34].

We emphasize that, due to the reduced dimensionality and its linearity, we do not expect the EES to be an accurate model of the original system. However, because of the geometrical constraints, it is possible to use the EES to (approximately) compute answers to a very limited set of questions about the system. Specifically, they are questions on gene expression levels when external changes are made within 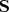. As an example, the EES can predict gene expression levels in double knockout mutants. We tested the predictions using previously published data on a double knockout mutant in an oxygen deprivation network of *E.coli*. (Here, as in most cases, the underlying network is unknown.) We identified the group of 299 genes to be studied using the Gene Ontology database. The EES was computed using the expression levels of five single knockout mutants, and used to predict their expression levels in the double mutant. The predictions were significantly different from the experimentally obtained expression levels for less than 30% of genes.

Interestingly, the EES can be used to compute how expression levels of genes within 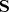 need to be changed so that the equilibrium of the entire network is moved from its initial state 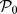 to, or as close as possible to, a pre-specified position 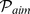. We showed through an example that the solution computed using the EES is close to that of the full network. However, the efficacy of the move depends on the proximity of 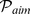 to the surface 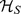. If 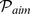 is far from 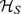, then the set of internal variables need to be expanded in order to find acceptable solutions.

Before concluding, we briefly address a few issues; the first is the observation that, in the parameter range considered, the model system given by Eqns. (1) and (4) have at most one stable steady state. Even though we required 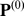 to be stable (by an appropriate choice of eigenvalues of the linearization), non-linear systems can, in general, be expected to have additional solutions. However, our model has a special feature: the signs of the partial derivatives 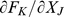 are independent of the state of the system. The analogous biological statement is that, if nodes 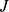 and 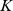 are isolated, the action of node 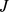 on node 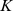 increases in magnitude as 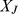 increases. Is this condition, combined with the choice of eigenvalues, sufficient to guarantee a uniques stable solution? We are currently studying this question. It should be noted that the uniqueness of solutions has been proven for several other classes of monotonic nonlinear systems [35]–[37].

The second issue involves the partitioning of genes into clusters and the choice of internal variables. Internal variables in the oxygen deprivation network of *E.coli* were already determined from the experiments reported in Ref. [32]. We used the GO classification to identify nodes belonging to the network. Different approaches can be used to partition genes into clusters when biological classifications are not available. For example, one could use topological (*e.g.*, persistent homology [38], [39]) or graph theoretic (*e.g.*, spectral clustering [23], community clustering [24]) methods. Integrated genomic analysis, which successfully identified subtypes of gliobastoma [40], can also be used in clustering genes through the use of heat maps [41], [42]. The choice of internal variables requires biological input. Mathematically, the requirement is that each node in the cluster can be affected by suitable changes in internal variables. As we mentioned, genes that translate to transcription factors, or microRNAs [28], [29] within the cluster, could act as internal nodes.

Third, can one estimate the proximity of 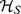 to the solution surface 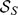
? Differences in gene expression levels of double knockout mutants are one measure of the proximity. Alternatively, we could use the corresponding differences in heterozygous single knockouts (whose expression levels are roughly half of the wildtype) and the predictions of the EES.

We believe that approaches similar to those outlined here can prove useful in treating complex genetic diseases by helping identify optimal combinations of up/down regulation of genes (or optimal combinations of single target drugs) that have minimal side effects and are most effective in moving the equilibrium of the network in its entirety to a preferred state. We hope our work motivates studies on this issue.

## Methods

### Construction of the EES

As illustrated in Figures 1, the EES is constructed so that, as internal variables are modified, the solutions of the system lie on the 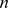-dimensional plane 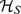. Thus the external variables are linear in 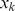
's, and consequently, have the form given by Eqns. (2). We need to compute the coefficients 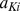 for 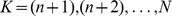 and 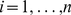. This is done by noting that the expression levels 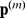 of each of the 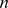 mutants 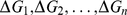 satisfies Eqn. (2), thus providing the conditions necessary to compute 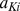
's.

We note, however, that the internal variables themselves are inter-related. For example, in the single knockout mutant 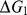, all expression levels (other than 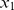) are determined by solving the last 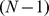 equations of (1). Thus, we need to derive relationships between the internal variables. Consider for example, the dependence of 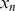 on the remaining internal variables. In order to find its form, let us reduce the set of internal variables to 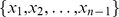; 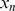 is now an external variable. Hence, with the approximations used in the paper, 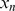 is a linear combination of the remaining internal variables. Since 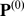 is one solution of the system
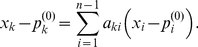
(6)Similar relationships are obtained for the other internal variables.

## Supporting Information

Supporting Information S1 Tables showing (1) The set G of 299 genes chosen to study the oxygen deprivation network of E.coli. These genes have the common biological function 0006355 “regulation of transcription, DNA-dependent” (2) Mean values of the 299 genes in the wildtype and the mutants. (3) Standard deviation of 299 genes in the wildtype and mutants. (4) The coefficients of the Effective Empirical Network. (5) Comparison between the predicted and experimental gene expression levels for the double knockout of fnr and arcA. The experimental data are normalized by the corresponding mean value of the wildtype replicates (item (2)).
(0.20 MB XLS)

## References

[1] Wagner A (2005). Robustness and Evolvability in Living Systems..

[2] Bernard C (1927). An Introduction to the Study of Experimental Medicine.

[3] Waddington C (1959). Canalization of Development and Genetic Assimilation of Acquired Characters.. Nature.

[4] Waddington C (1960). Experiments on Canalizing Selection.. Genetical Research.

[5] Hecker M, Lambeck S, Toepfer S, van Someren E, Guthke R (2009). Gene regulatory network inference: Data integration in dynamic models-A review.. Biosystems.

[6] Barabasi A, Oltvai Z (2004). Network biology: Understanding the Cell's Functional Organization.. Nature Reviews Genetics.

[7] Ravasz E, Somera A, Mongru D, Oltvai Z, Barabasi A (2002). Hierarchical Organization of Modularity in Metabolic Networks.. Science.

[8] Oltvai Z, Barabasi A (2002). Life's Complexity Pyramid.. Science.

[9] Kitano H (2007). Towards a Theory of Biological Robustness.. Molecular Systems Biology.

[10] Hauser K, Abdollahi A, Huber PE (2009). Inverse system perturbations as a new methodology for identifying transcriptomic signaling participants in balanced biological processes.. Cell Cycle.

[11] Zimmermann GR, Lehar J, Keith CT (2007). Multi-target therapeutics: when the whole is greater than the sum of the parts.. Drug Discovery Today.

[12] Frantz S (2005). Playing Dirty.. Nature.

[13] Yang K, Bai H, Ouyang Q, Lai L, Tang C (2008). Finding multiple target optimal intervention in disease-related molecular network.. Molecular Systems Biology.

[14] Gardner TS, di Bernardo D, Lorenz D, Collins JJ (2003). Inferring genetic networks and identifying compound mode of action via expression profiling.. Science.

[15] Bornholdt S (2005). Less is More in Modeling Large Genetic Networks.. Science.

[16] Covert MW, Palsson BO (2003). Constraints-based models: Regulation of gene expression reduces the steady-state solution space.. Journal of Theoretical Biology.

[17] Milo R, Shen-Orr S, Itzkovitz S, Kashtan N, Chklovskii D (2002). Network motifs: Simple building blocks of complex networks.. Science.

[18] Shen-Orr S, Milo R, Mangan S, Alon U (2002). Network motifs in the transcriptional regulation network of Escherichia coli.. Nature Genetics.

[19] Bornholdt S (2008). Boolean network models of cellular regulation: prospects and limitations.. Journal of the Royal Society Interface.

[20] Faith JJ, Hayete B, Thaden JT, Mogno I, Wierzbowski J (2007). Large-scale mapping and validation of escherichia coli transcriptional regulation from a compendium of expression profiles.. PLOS Biology.

[21] Someren EPv, Wessels LFA, Reinders MJT (2000). Linear modeling of genetic networks from experimental data.. Proceedings of the Eighth International Conference on Intelligent Systems for Molecular Biology.

[22] di Bernardo D, Thompson M, Gardner T, Chobot S, Eastwood E (2005). Chemogenomic profiling on a genomewide scale using reverse-engineered gene networks.. Nature Biotechnology.

[23] von Luxburg U (2007). A tutorial on spectral clustering.. Statistics and Computing.

[24] Newman MEJ (2004). Fast algorithm for detecting community structure in networks.. Physical Review E.

[25] Sturn A, Quackenbush J, Trajanoski Z (2002). Genesis: Cluster Analysis of Microarray Data.. Bioinformatics.

[26] Babu M, Luscombe N, Aravind L, Gerstein M, Teichmann S (2004). Structure and Evolution of Transcriptional Regulatory Networks.. Current Opinion In Structural Biology.

[27] Gill G (2001). Regulation of the initiayion of eukaryotic transcription.. Essays in Biochemistry.

[28] Ambros V (2004). The functions of animal microRNAs.. Nature.

[29] Bartel D (2004). MicroRNAs: Genomics, biogenesis, mechanism, and function.. Cell.

[30] Herrgard MJ, Covert MW, Palsson BO (2003). Reconciling gene expression data with known genome-scale regulatory network structures.. Genome Research.

[31] Salmon KA, Hung S, Steffen NR, Krupp R, Baldi P (2005). Global gene expression profiling in escherichia coli k12 - effects of oxygen availability and arca.. Journal of Biological Chemistry.

[32] Covert MW, Knight EM, Reed JL, Herrgard MJ, Palsson BO (2004). Integrating high-throughput and computational data elucidates bacterial networks.. Nature.

[33] Tsukakoshi M, Kurata S, Nomiya Y, Ikawa Y, Kasuya T (1984). A Novel Method of DNA Transfection by Laser Microbeam Cell Surgery.. Applied Physics B-Photophysics and Laser Chemistry.

[34] Bertram J (2006). MATra - Magnet Assisted Transfection: Combining Nanotechnology and Magnetic Forces to Improve Intracellular Delivery of Nucleic Acids.. Current Pharmaceutical Biotechnology.

[35] Feinberg M (1995). The existence and uniqueness of steady states for a class of chemical reaction networks.. Arch Rational Mech Anal.

[36] Hirsch MW, Smith H (2005). Monotone dynamical systems.. Handbook of differential equations: ordinary differential equations.

[37] Angeli D, Sontag ED (2008). Translation-invariant monotone systems, and a global convergence result for enzymatic futile cycles.. Nonlinear Anal Real World Appl.

[38] Carlsson G, Zomorodian A (2009). The Theory of Multidimensional Persistence.. Discrete & Computational Geometry.

[39] Carlsson G (2009). Topology and Data.. Bulletin of the American Mathematical Society.

[40] Verhaak RGW, Hoadley KA, Purdom E, Wang V, Qi Y (2010). Integrated Genomic Analysis Identifies Clinically Relevant Subtypes of Glioblastoma Characterized by Abnormalities in PDGFRA, IDH1, EGFR, and NF1.. Cancer Cell.

[41] Eisen M, Spellman P, Brown P, Botstein D (1998). Cluster analysis and display of genome-wide expression patterns.. Proceedings of the National Academy of Sciences of the United States Of America.

[42] Spellman P, Sherlock G, Zhang M, Iyer V, Anders K (1998). Comprehensive identification of cell cycle-regulated genes of the yeast Saccharomyces cerevisiae by microarray hybridization.. Molecular Biology of the Cell.

